# Coronary Stent for Right Transverse Venous Sinus Stenosis in a Patient With Symptomatic Idiopathic Intracranial Hypertension

**DOI:** 10.7759/cureus.36073

**Published:** 2023-03-13

**Authors:** Camilo A Perdomo Luna, Laura Campaña Perilla, José D Cardona, Enrique Jimenez-Hakim, Juan Andres Mejía

**Affiliations:** 1 Radiology, University Hospital Fundación Santa Fe de Bogotá, Bogotá, COL; 2 Radiology, University Hospital Fundación Santa Fé de Bogotá, Bogotá, COL; 3 Neurosurgery, Fundación Santa Fe de Bogotá, Bogotá, COL; 4 Radiology, Fundación Santa Fe de Bogotá, Bogotá, COL

**Keywords:** coronary stent, endovascular procedure, venous sinus stenosis, idiopathic intracranial hypertension, neurointerventional radiology

## Abstract

A 59-year-old woman was referred to the neuro-interventional team with complaints of headache, papilledema, and visual disturbances. Imaging and Lumbar puncture revealed signs consistent with idiopathic intracranial hypertension with stenosis of the right transverse venous sinus. The neurosurgery board chose to treat her with an endovascular approach and stenting. During the procedure, the right jugular vein revealed decreased blood flow. This led to a left jugular vein access through the confluence of venous dural sinuses. However, an incomplete confluence required the catheter to ascend the superior sagittal sinus before descending to the right transverse sinus. The carotid catheter system kept herniating up the SSS, risking rupture. Given the intraoperative findings and the available equipment, a more flexible coronary catheter system was chosen. This catheter device allowed plasty and successful stent deployment. A lumbar puncture was performed, and the patient was discharged. A follow-up MRI at five weeks showed signs of intracranial hypertension improvement and the patient reported Improvement in symptoms. To our knowledge, this is the first time this type of device has been used in this anatomical location for this pathology.

## Introduction

Idiopathic intracranial hypertension (IIH) is a condition of elevated cerebrospinal fluid (CSF) pressures, signs and symptoms of increased intracranial pressure (ICP), no localizing or focal neurologic signs or impaired level of consciousness, and no etiology for increased ICP on neuroimaging findings, as defined by the Dandy Criteria. Signs and symptoms include headache, papilledema, visual field loss, transient visual obscuration, and pulse-synchronous tinnitus. The maximum normal CSF pressure is usually around 15 to 20 depending on the literature [1].

Neuroimaging findings that may indicate a diagnosis of IIH include an empty sella turcica, slit-like ventricles, posterior scleral flattening, distension of the optic nerve sheath, enhancement of the optic nerve head, tortuosity of the optic nerves, and prominence of Meckel's cave [2]. It can also present with venous sinus stenosis (VSS) leading to obstruction to flow and higher-pressure gradients within the dural venous sinus system [3].

IIH has a significant predisposition toward obese women of reproductive age, and the population incidence is rising in tandem with the global prevalence of obesity [4]. Recent research showing the pathogenic involvement of metabolic and hormonal variables has led to the identification of several pharmacological targets and the development of innovative treatment medicines. Depending on the patient’s characteristics, treatment consists of a combination of weight loss, acetazolamide, avoidance of precipitating factors, and surgery [5]. Advancements in the field of endovascular procedures made venous sinus stenting, alternate therapy. Currently used stents and catheter systems have characteristics that may not be optimal for patients with anatomical variants. Further data are needed about different types of stents and catheter systems for the treatment of IHH with VSS.

## Case presentation

A 59-year-old woman with a 30-year history of well-controlled migraine developed a progressively worsening, constant, bilateral headache with photophobia, phonophobia, tinnitus, and memory loss. Eight months after the onset of symptoms, she consulted a neurologist who ordered a lumbar puncture (LP) and a 1.5T non-contrast brain MRI with T1, T2, diffusion, and susceptibility sequences with an initial opening pressure of 28 cm of H_2_O and 20 cm of H_2_O after CSF drainage. Overall, her symptoms and imaging were compatible with IIH and stenotic right transverse venous sinus that had significant repercussions on intra and extracranial structures and explained the patient’s clinical presentation. Her case was evaluated by the Neurosurgery Board on July 6, 2022. There it was decided that her treatment should be endovascular management with endoprosthesis (stent) as it would aid in restoring and remodeling cerebral venous Dural structures. This therapy would significantly improve the patient's symptoms like headaches photophobia or tinnitus.

**Figure 1:**
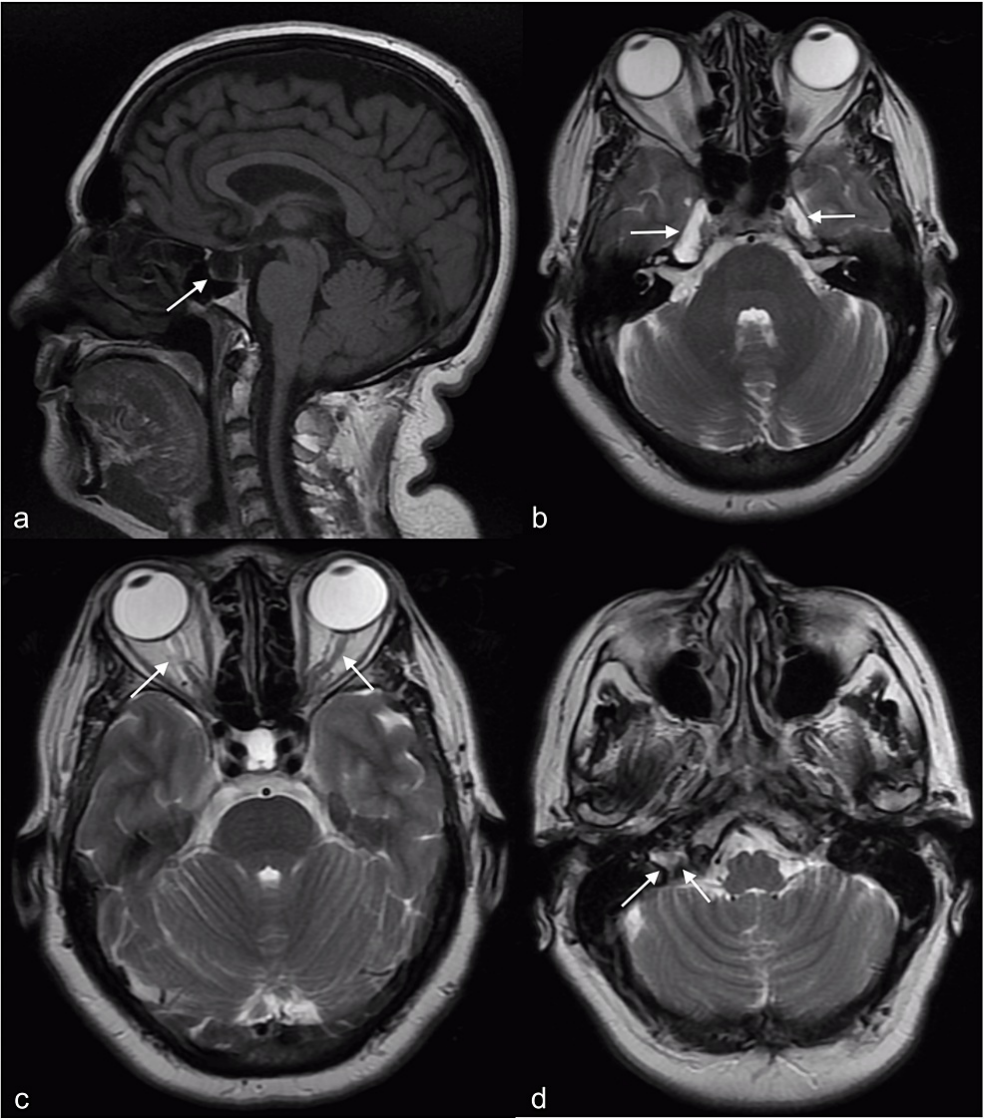
Magnetic resonance imaging of the brain shows indirect signs of intracranial hypertension. (a) Sagittal view showing empty Sella turcica (white arrow). (b) Axial view showing dilation of Meckel's cavum (white arrows). (c) Axial view showing thickening of the optic nerve sheath (white arrows). (d) Axial view showing chronic occlusion of the right internal jugular vein (white arrows).

The MRI on presentation showed signs consistent with IIH, empty Sella turcica, thickening of the optic disc, dilation of Meckel's cavum, flattening of the posterior eye globe with thickening of the optic nerve sheath, and chronic occlusion of the right internal jugular vein (Figures 1a-1d). The neurology team performed an LP with opening pressure (OP) of 28 cm of H_2_O confirming the diagnosis of IIH. Gadolinium-enhanced MRI confirmed the previous findings and magnetic resonance venography with 3D reconstruction showed right transverse VSS.

Using the right femoral arterial approach, an arteriographic study of the intracranial and extracranial carotid and vertebrobasilar vascular axes was performed. A hemostatic valve from the right cervical internal carotid was used for arterial control. Subsequently, the right femoral vein was punctured for the advancement of the 8F femoral introducer, through which a Neuron MAX® 088 (Penumbra Inc., USA) was navigated with an internal Select Vert 5F assisted with a 180cm hydrophilic guidewire (Penumbra, USA). A triaxial configuration was performed with a SOFIA™ 6F (Microvention, USA) distal access catheter.

**Figure 2:**
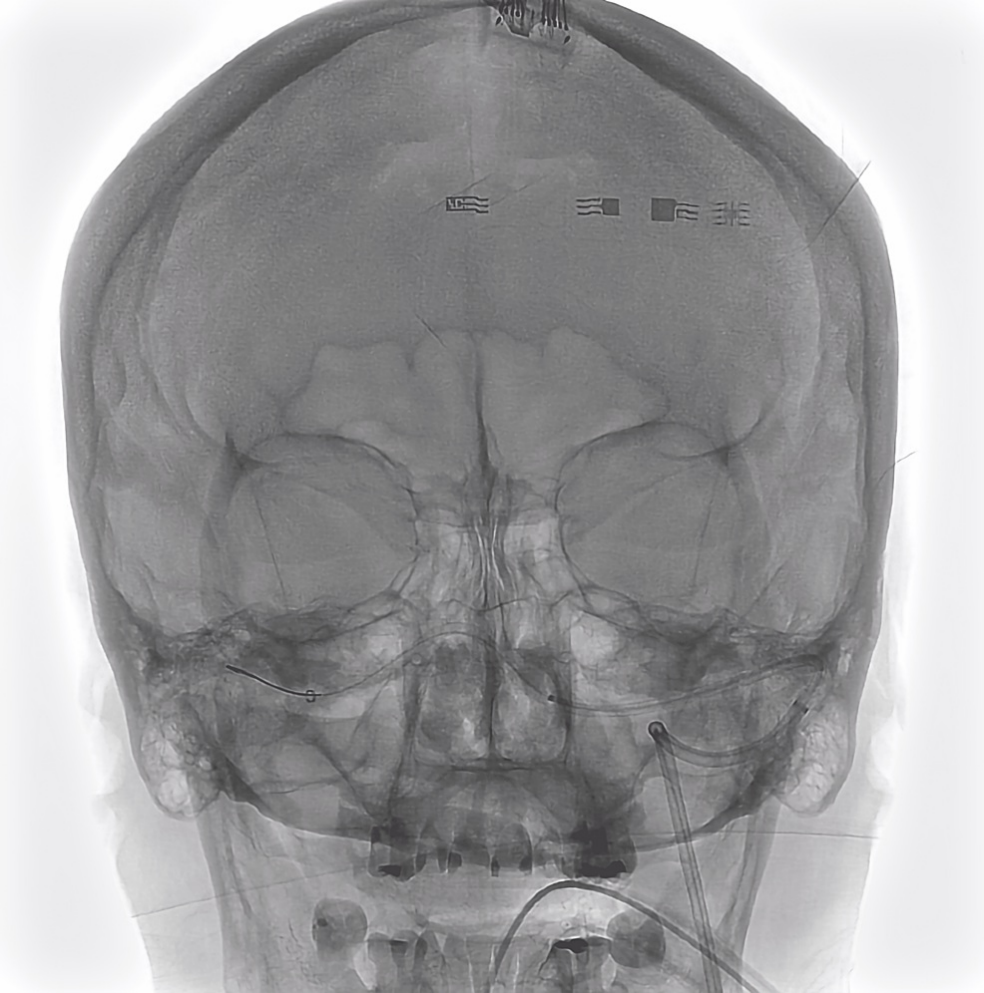
Approach through the left jugular vein to access the right transverse venous sinus.

The procedure was technically difficult due to chronic occlusion of the right internal jugular vein, requiring access through the left internal jugular vein. Due to the incomplete confluence of the dural sinuses (Figure 2), it was necessary to ascend to the sagittal sinus and subsequently descend to the right transverse sinus with a PROGREAT® (Terumo, Japan) microcatheter, as support and for measurement of the pressure gradient. The WALLSTENT™ 7x30mm stent (Boston Scientific, USA) was advanced and assisted by a Transend™ 0.014x200cm (Boston Scientific, USA) micro guide. Due to oversizing and curvature of the torcula, the stent herniated toward the superior sagittal sinus (SSS) putting the structure at risk for dissection and rupture.

**Figure 3:**
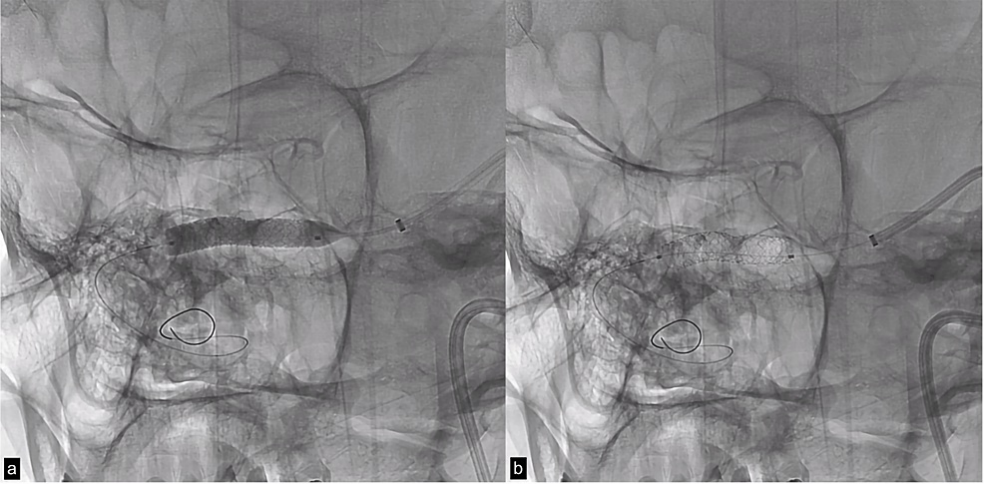
(a) Stent deployment after plasty with sterling balloon and (b) stenting with endoprosthesis mounted on an onyx balloon.

**Figure 4:**
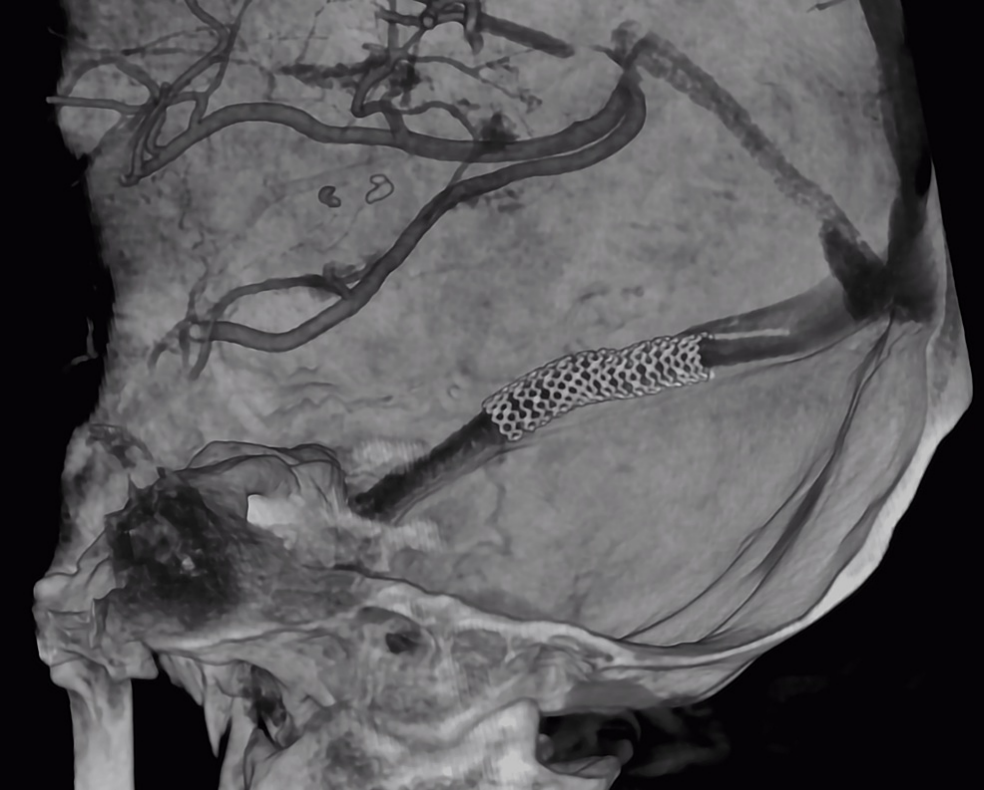
3D reconstruction of the right transverse venous sinus with the stent in situ.

**Figure 5:**
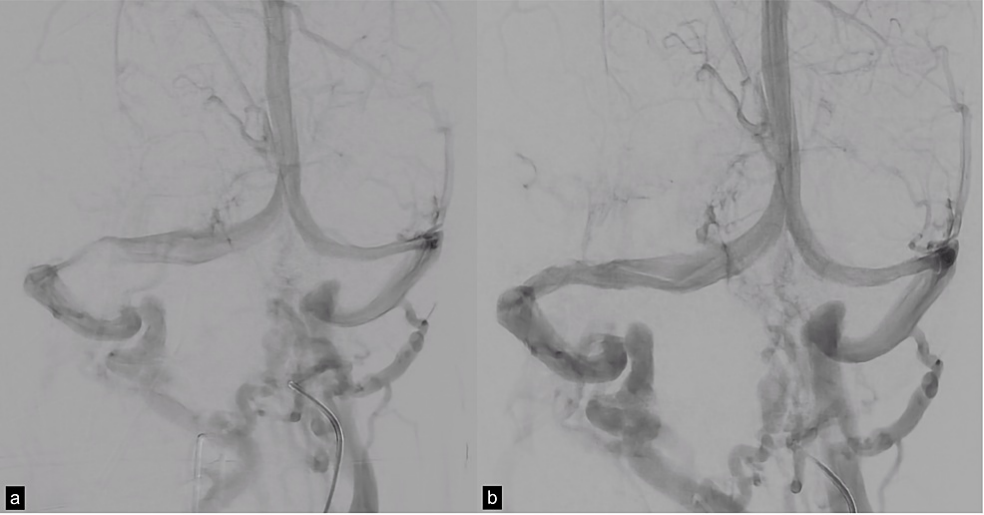
(a) Right transverse sinus stenosis prior to stent placement. (b) Flow improvement after stent placement.

Considering these findings, the carotid catheter system was replaced by a Navien™ 072 (Medtronic, USA) with Streaming micro guide 0.014 (Asahi Intecc, Japan) for the advancement of a balloon-mounted coronary stent Resolute Onyx™ 5.0x26mm balloon (Medtronic, USA) with which complete patency of the transverse sinus was achieved (Figures 3a, 3b, 4). After stent insertion, the permeability of the right transverse sinus improved (Figures 5a, 5b). The patient was started on dual antiplatelet therapy with clopidogrel 75 mg and acetyl-salicylic acid 100mg per day and transferred to the intensive care unit.

The day after the procedure, diagnostic/therapeutic LP was performed. OP was initially 25 cmH_2_O and 15 cm of H_2_O after drainage. Five weeks after stent placement, a contrasted MRA displayed changes toward normalization of the circumference of the eyeballs, bilateral decrease in amplitude of optic nerve sheath, and improvement in right transverse sinus caliber and flow signal. These findings are consistent with the improvement of papilledema.

## Discussion

IIH is characterized by tinnitus, headache, visual disturbances, papilledema, and elevated CSF opening pressure on LP in the absence of an intracranial mass [6,7]. Most patients with this pathology present with stenosis at the sigmoid-transverse sinus junction [8]. Studies have further suggested an obstruction to flow leading to significantly higher-pressure gradients in the superior sagittal and transverse sinuses [3]. The mainstay treatment of IIH is weight loss plus acetazolamide, 10% of patients are resistant to this regimen and require surgery [5]. Surgical treatment consists of invasive techniques such as ventricular peritoneal shunt and optic nerve sheath fenestration [9]. Recently, venous sinus stenting has been proposed as an alternative therapy for IIH, and nowadays hundreds of patients have been successfully intervened and treated with this approach [6]. In 2017, a meta-analysis by Dinkin and Patsalides that included 282 patients found stenting effectively in reducing ICP and treating ICP-related symptoms, especially in the case of intrinsic VSS [9].

Currently, the types of stents deployed for VSS include stents intended for the biliary system, carotid, femoral and iliac arteries. These stents are stiffer and require larger deployment catheter systems, which is the opposite of what an ideal system would be for this region of the body, especially in patients with anatomical variants [9].

In this case, a patient with symptoms consistent with IIH, confirmed by imaging and LP was initially supposed to undergo stenting with a carotid stent and catheter system. During the procedure, the decreased venous flow was noted in the right jugular vein. This finding led to a left jugular vein approach via the confluence of venous dural sinuses. However, an incomplete confluence demanded the catheter to be advanced up the superior sagittal sinus, before coming down to the right transverse sinus. The carotid catheter system kept herniating up the SSS, putting the structure at risk of rupture. A switch to a more flexible catheter system was decided, picking a coronary catheter system as a better option. As it turned out, this catheter system permitted successful plasty and stent deployment.

This report describes the successful treatment of a patient with symptomatic IIH with transverse sinus stenosis that required the deployment of a cardiac endovascular stent. To our knowledge, this is the first time a coronary catheter system has been used to deploy a stent in VSS.

## Conclusions

Management for patients with IIH secondary to VSS can be aided by endovascular stenting by restoring venous flow and improving the patient's symptoms. Traditional lifestyle and pharmacological treatment methods have been useful, but there are patients for whom these treatments are refractory and require more invasive procedures. Traditionally, endovascular treatment of IIH with VSS is performed using the carotid, iliac, femoral, or biliary systems. Here we show the first case reported in the literature of using a coronary stent to manage IIH as an alternative in patients with complex anatomies. We believe this report may serve to spark and encourage further research on the use of alternative devices for VSS in patients with complex anatomies.
